# Real-time feedback on chest compression efficacy by hands-free carotid Doppler in a porcine model

**DOI:** 10.1016/j.resplu.2024.100583

**Published:** 2024-02-20

**Authors:** Bjørn Ove Faldaas, Erik Waage Nielsen, Benjamin Stage Storm, Knut Tore Lappegård, Bent Aksel Nilsen, Gabriel Kiss, Eirik Skogvoll, Hans Torp, Charlotte Björk Ingul

**Affiliations:** a Faculty of Nursing and Health Sciences, Nord University, Bodø, Norway; b Department of Circulation and Medical Imaging, Norwegian University of Science and Technology (NTNU), Trondheim, Norway; c Department of Clinical Medicine, Faculty of Health Sciences, UiT the Arctic University of Norway, Tromsø, Norway; d Department of Surgery, Nordland Hospital Trust, Bodø, Norway; e Department of Pain Management and Research, Division of Emergencies and Critical Care, Oslo University Hospital, Oslo, Norway; f Department of Medicine, Nordland Hospital Trust, Bodø, Norway; g Department of Computer Science (IDI), Faculty of Information Technology and Electrical Engineering, Norwegian University of Science and Technology (NTNU), Trondheim, Norway; h Clinic of Anesthesia and Intensive Care Medicine, St Olav University Hospital, Trondheim, Norway

**Keywords:** Cardiopulmonary resuscitation (CPR), Manual chest compressions, Doppler ultrasound, Carotid artery, Real-time feedback, Physiologic monitoring

## Abstract

**Aim:**

Current guidelines for cardiopulmonary resuscitation (CPR) recommend a one-size-fits-all approach in relation to the positioning of chest compressions. We recently developed RescueDoppler, a hands-free Doppler ultrasound device for continuous monitoring of carotid blood flow velocity during CPR. The aim of the present study is to investigate whether RescueDoppler via real-time hemodynamic feedback, could identify both optimal and suboptimal compression positions.

**Methods:**

In this model of animal cardiac arrest, we induced ventricular fibrillation in five domestic pigs. Manual chest compressions were performed for ten seconds at three different positions on the sternum in random order and repeated six times. We analysed Time Average Velocity (TAV) with chest compression position as a fixed effect and animal, position, and sequential time within animals as random effects. Furthermore, we compared TAV to invasive blood pressure from the contralateral carotid artery.

**Results:**

We were able to detect changes in TAV when altering positions. The positions with the highest (range 19 to 48 cm/s) and lowest (6–25 cm/s) TAV were identified in all animals, with corresponding peak pressure 50–81 mmHg, and 46–64 mmHg, respectively. Blood flow velocity was, on average, highest at the middle position (TAV 33 cm/s), but with significant variability between animals (SD 2.8) and positions within the same animal (SD 9.3).

**Conclusion:**

RescueDoppler detected TAV changes during CPR with alternating chest compression positions, identifying the position yielding maximal TAV. Future clinical studies should investigate if RescueDoppler can be used as a real-time hemodynamical feedback device to guide compression position.

## Introduction

High-quality chest compressions are needed to maintain adequate blood flow and oxygen delivery to vital organs during cardiopulmonary resuscitation (CPR).

According to the 2021 European Resuscitation Guidelines (ERC), chest compressions should be performed with a rate ranging from 100 to 120 compressions per minute and a depth of 5 to 6 cm and complete chest recoil after each compression.^1^ Despite adhering to guidelines, external manual chest compressions are inherently inefficient, delivering only 30% to 40% of normal blood flow to the brain and less than one-third of normal blood flow to the heart.^2^

ERC and The International Liaison Committee on Resuscitation (ILCOR) recommend administering compressions at the lower half of the sternum.^1^, ^3^, ^4^, ^5^ However, research has shown that the optimal compression position varies among adult patients.^6^, ^7^, ^8^ Individual patient anatomy, including the position of the left ventricle in relation to the anterior-posterior axis, and the location of the heart within the thoracic cavity,^6^, ^9^, ^10^, ^11^ potentially influences the effectiveness of chest compressions.^8^, ^12^, ^13^, ^14^, ^15^, ^16^, ^17^ Targeted compression of the left ventricle is suggested to improve hemodynamics,^18^, ^19^, ^20^ but the optimal chest compression position is a matter of debate.^10^, ^21^, ^22^ Suboptimal hand placement during CPR can therefore, significantly diminish its effectiveness: if compressions are performed over the left ventricular outflow tract, this can lead to obstruction of left ventricular ejection and causing insufficient blood supply to vital organs.^6^, ^17^, ^23^, ^24^, ^25^

Even chest compressions performed by health professionals may be of sub-optimal quality, as rescuers often overestimate compression depth, and underestimate rate.^26^ Further, chest compression quality is reduced when the rescuers become exhausted during prolonged CPR.^26^

Automated real-time feedback devices are promising tools for enhancing the acquisition and retention of CPR skills, thus improving the overall quality of CPR.^26^ ILCOR recommends utilizing feedback devices during CPR training, which offer specific guidance on hand placement, compression rate, depth and release (low-certainty evidence, weak recommendation).^4^ Real-time processing of corrective feedback data results in the generation of visual information or auditory cues, such as voice messages or tones, enabling rescuers to quickly adjust their CPR technique to adhere to CPR guidelines.^27^ However, these devices only measure the rescuer performing CPR.

Available devices include metronomes, audio-visual devices, and smartphone apps. Currently, there is a lack of real-time non-invasive hemodynamic measurement methods that can assess the effectiveness of compressions in the patient.

We recently developed the RescueDoppler, a non-invasive hands-free Doppler system that continuously detects blood flow in the carotid artery.^28^ In a porcine model, RescueDoppler reliably identifies pulsative blood flow at blood pressures below 60 mmHg.^28^

The aim of the present study is to investigate whether RescueDoppler, via real-time hemodynamic feedback, can identify variations in TAV between chest compression positions and identify optimal as well as suboptimal positions.

## Methods

The study was conducted using five male domestic pigs (sus scrofa domesticus) obtained from a certified pathogen-free farm, using our previously described animal model.^28^ The Norwegian Animal Research Authority approved the study (FOTS-ID 25415). The experiments were conducted in accordance with the “*Regulations on Animal Experimentation”* set forth by the Norwegian Animal Welfare Act^29^ and the Animal Research: Reporting of In Vivo Experiments (ARRIVE) guidelines.^30^

### Animal preparation

At the farm, animals were sedated with ketamine, atropine, and midazolam before a short transport to the lab. The animals were anesthetized using midazolam and morphine injected through an ear vein. Anaesthesia was maintained using morphine, midazolam, and thiopental infusions. The animals were ventilated using a GE Engstøm Carestation Ventilator (GE Healthcare), with volume-controlled ventilation at an FiO2 (fraction of inspired oxygen) of 0.21, a tidal volume of 10–15 mL/kg, and a respiratory rate of 13–16 bpm. Continuous 5-lead ECG, continuous invasive, arterial- and central venous pressures, and end-tidal CO2 (ETCO2) were continuously monitored, and minute ventilation was adjusted to maintain a pH level of 7.4.^28^ To ensure optimal positioning and immobilization during chest compressions, the animals were placed on a vacuum mattress,^31^ and kept normothermic (38.5–39.0 °C).

### Instrumentation

Ultrasound-guided Seldinger's technique was used to insert an 8-Fr Avanti + Sheath introducer (Cordis, Santa Clara, CA) into the left external jugular vein and a 4-Fr, 8-cm Leadercath arterial catheter (Vygon Ltd., Swindon, UK) into the left internal carotid artery. Instrumentation has been described in detail previously.^28^ An ICD (St. Jude Medial Ellipse DR Model CD2377-36C, Merlin Patient Care System, Abbott, Sylmar, CA, USA) was used to induce ventricular fibrillation and for subsequent defibrillation.^28^ The ICD lead was introduced via the external jugular vein into the right ventricle under echocardiographic supervision (GE Vivid S7 Pro, 3S probe. GE Vingmed, Horten, Norway).^28^ The ICD device was implanted subcutaneously at the upper right part of the chest. To initiate ventricular fibrillation, we employed the DC Fibber induction technique using the ICD and St. Jude Medical's software as earlier described.^28^ Defibrillation was done with 30 joule using the ICD. To avoid hypoxia, inspired oxygen concentration was increased to 100% before and after each sequence.

### Monitoring and data recording

All data were recorded using a Phillips IntelliVue Patient Monitor MP70 (Philips Medizin Systeme, Boeblingen, Germany) and stored using proprietary software.

The RescueDoppler probe was positioned over the right carotid artery and fixed with adhesive tape. We integrated an accelerometer onto a custom-moulded plate (100 mm diameter, 30 mm height) to record chest compression depth and rate, and then transmitted these signals to the RescueDoppler system for post-analysis. Time Average Velocity (TAV) from the RescueDoppler signal was calculated using MATLAB software (MathWorks for Apple, R2021a). Data were exported to Microsoft Excel (Excel for Mac version 16.76, Microsoft Corp., stat) for further analysis.

### RescueDoppler system

The RescueDoppler system, previously described in detail,^28^ is a non-image pulsed wave Doppler, consisting of a custom-made carotid ultrasound Doppler probe, a dedicated scanner, and a laptop equipped with a MATLAB-based user interface that provides real-time visualization. This system incorporates two transducers housed within a 3D-printed casing set at a fixed angle of ±30°. Each transducer has an unfocused aperture measuring 30 × 6 mm and operates at a central frequency of 4 MHz Utilizing digital signal processing, this advanced technology employs 32 depth ranges, equally distributed between 8 and 45 mm. The depth ranges are visualized as a colour M−mode, with a Doppler spectrogram detailing the chosen range and sample volume. The maximum velocity curve was automatically traced from the spectrogram. Angle correction was performed based on the velocity curve from each transducer. Wall and clutter filters were added in the post-processing to remove wall motion noise from being registered. All Doppler velocities below 30 cm/s were interpreted as arising from tissue movements and thus changed to 0 cm/s. Noise-generating velocity spikes were removed by a digital filter.

### Cardiopulmonary resuscitation model

**Fig. 1:**
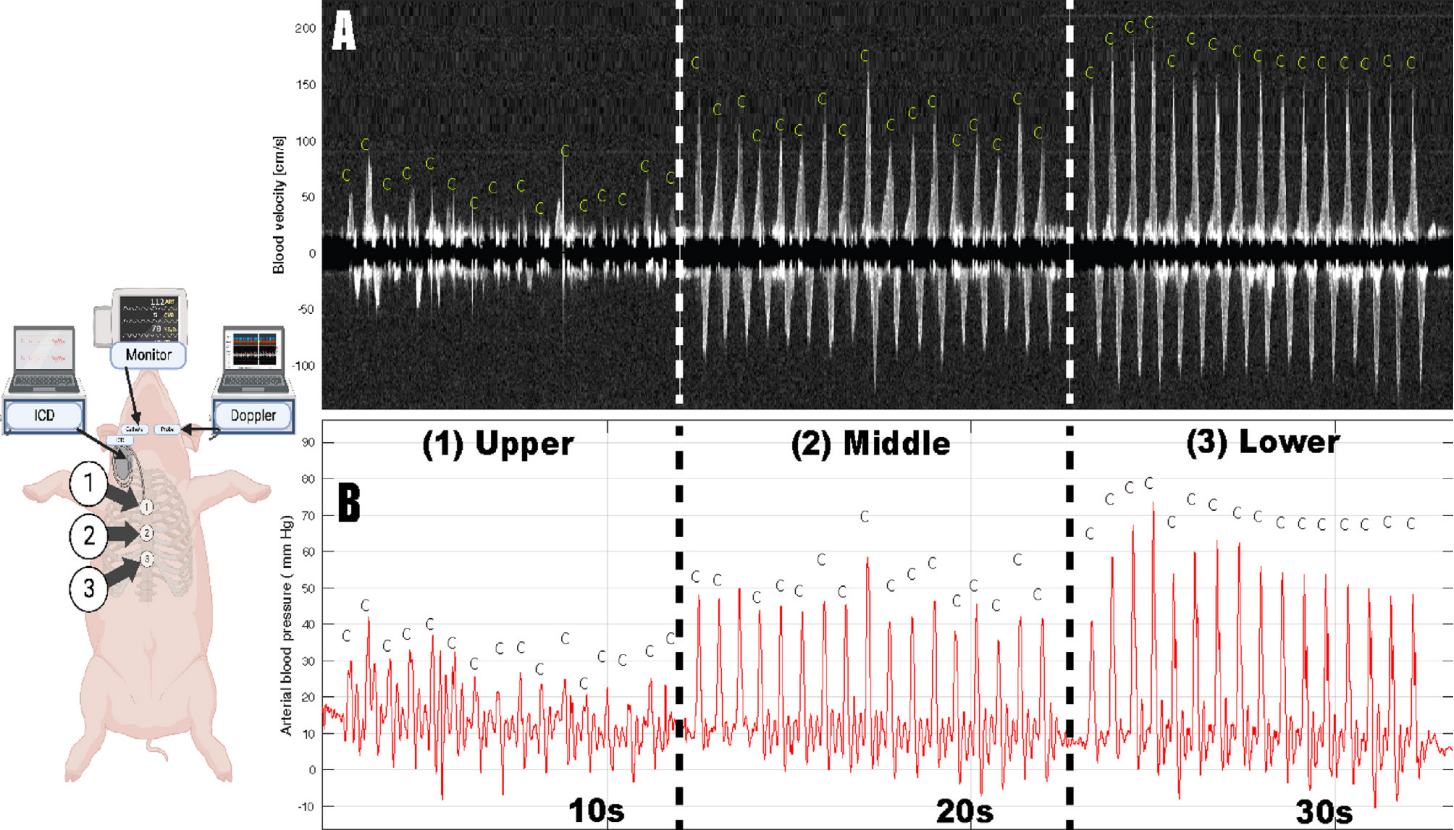
Chest compressions sequence illustration. Left: Animal model illustrating hand placement positions during chest compressions and placement of implantable cardioverter-defibrillator, monitor and RescueDoppler. Hand position 1: Over the upper sternum. Position 2: Over the lower third of the sternum. Position 3: At the xiphoid process. The distance between positions was 5 cm. Right: Upper panel (A): Left carotid Doppler spectrogram. Lower panel (B): Invasive right carotid blood pressure curve. A sequence of chest compressions at three distinct positions –upper, middle, and lower (animal illustration) are shown. Compressions (100 compressions per minute) lasted 10 seconds at each position. Animal illustration created with Biorender.com.

The model was developed based on recommendations for porcine CPR models^31^, ^32^, ^33^ and ERC guidelines.^1^, ^32^ Three chest compression positions on the sternum, five cm apart, were marked with a waterproof marker pen. The “Upper” position was located at the superior part of the sternum, the “Middle” position at the lower third of the sternum, and the “Lower” position at the xiphoid process (Fig. 1). A single rescuer conducted manual chest compressions at a rate of 100 compressions per minute, guided by a metronome, for ten seconds at each position across all animals, while remaining blinded to all hemodynamic measurements.^34^ A sequence consisted of chest compressions that lasted for 10 seconds at each position with a total duration of 30 seconds. The chest compression positions were assigned in random order: position 2–1–3, 1–2–3, 3–2–1, 1–3–2, 3–1–2, 2–3–1 (Fig. 1). After each sequence, the ICD delivered an unsynchronized shock to restore sinus rhythm. Between sequences, the pigs were given a five-minute recovery time to restore normal vital parameters.

### Statistics

Descriptive statistics include the mean with standard deviation (SD) or median with range, as appropriate. We entered TAV as the outcome variable in a linear mixed model, with chest compression position on the sternum (upper, middle, lower) as a fixed (categorical) effect, and animal identity, position and sequential time within animals as random effects using the method of restricted maximum likelihood (REML).

We then investigated whether the individual sequences were consistent with the overall results in each animal. This was done by ranking the compression positions according to TAV (best, intermediate, worst) in all six sequences from each animal, and cross tabulating them with the average ranking from the same animal.

Descriptive statistical analyses were obtained with IBM SPSS Statistics for MAC version 27.0.1.0 (Armonk, NY: IBM Corp). MATLAB, with an in-house software program, was employed to display and analyse the Doppler spectrograms. Stata version 17 (StataCorp, College Station, Texas, USA) with the mixed command was employed for the linear mixed model.

## Results

### Chest compression data

Doppler and blood pressure data from the five male animals and 29 out of 30 CPR sequences were included. One sequence was excluded due to suboptimal contact between the probe and the skin. Compression depth data were available for 3 out of 5 animals and 12 out of 29 sequences (Supplementary Table 1). Compression depth data for animal 1 are presented in Supplementary Fig. 1. The variation in mean compression depth among positions for animal 1 was 0.2 cm (Upper 4.0 cm, Middle 4.2 cm, Lower 4.0 cm) (Supplementary Table 1). The mean compression depth for positions across three animals and 12 sequences was: upper 3.7 cm, middle 3.9 cm, and lower 4.0 cm. We excluded the first two compressions of each sequence to reduce artefacts resulting from changing the compression position. Baseline hemodynamic for animals (n = 5), mean systolic blood pressure 111 mmHg (range: 96–129 mmHg) mean diastolic blood pressure 69 mmHg (range: 57–82 mmHg). Descriptive statistics for animals are shown in Table 1.

**Table 1:** Baseline characteristics of animals.

	Weight (kg)	Thorax diameter (cm)	Carotid Diameter (mm)	Carotid Depth (mm)	MAP (mmHg)	TAV (cm/s)
N = 5						
Mean	31.2	67.0	4.3	22.2	87.4	22.6
Std. Deviation	0.8	1.3	0.5	27.7	13.9	6.5
Range	30–32	66–69	3.7–5.0	19–25	70–104	17–31

Std. Deviation: Standard deviation, MAP: Mean Arterial Pressure, TAV: Time Averaged Velocity.

### Chest compression position

TAV and mean arterial blood pressure (MAP) corresponding to each chest compression position for all animals, along with the individual measurements in each position and sequence are shown in Table 2.

**Table 2:** Time Average Velocity and Mean Arterial Pressure for chest compression positions.

Animal			1	2	3	4	5	ALL
Position								
**Upper**	TAV (cm/s)	Mean	6	12	23	27	9	15.3
		Std. Deviation	3.2	7.6	18.2	12.8	9.1	
		Minimum	1	3	2	8	0	
		Maximum	10	24	45	42	20	
		Range	9	20	43	34	19	
		N	6	6	5	6	6	
	MAP (mmHg)	Mean	13	24	26	30	33	25.1
		Std. Deviation	1.7	1.9	1.8	5.1	5.2	
		Minimum	10	21	24	24	25	
		Maximum	15	26	28	37	38	
		Range	5	5	5	13	14	
		N	6	6	5	6	6	
**Middle**	TAV (cm/s)	Mean	36	48	31	32	19	33.1
		Std. Deviation	9.7	17.1	20.0	5.7	4.8	
		Minimum	20	20	6	24	13	
		Maximum	46	65	52	38	26	
		Range	26	45	45	14	13	
		N	6	6	5	6	6	
	MAP (mmHg)	Mean	21	26	28	33	35	28.8
		Std. Deviation	5.7	2.7	0.8	8.1	6.2	
		Minimum	15	23	27	28	31	
		Maximum	28	30	29	50	46	
		Range	13	7	2	21	15	
		N	6	6	5	6	6	
**Lower**	TAV (cm/s)	Mean	39	36	21	25	14	27.2
		Std. Deviation	8.5	6.0	12.7	3.2	5.3	
		Minimum	28	28	9	21	9	
		Maximum	48	43	37	29	24	
		Range	20	16	28	8	14	
		N	6	6	5	6	6	
	MAP (mmHg)	Mean	21	23	27	32	32	27.0
		Std. Deviation	5.2	1.7	2.0	7.0	3.3	
		Minimum	15	20	24	25	27	
		Maximum	28	24	29	43	36	
		Range	13	4	5	18	9	
		N	6	6	5	6	6	

TAV; Time Average Velocity, MAP; Mean Arterial Pressure and Std. Deviation; Standard Deviation.

**Fig. 2:**
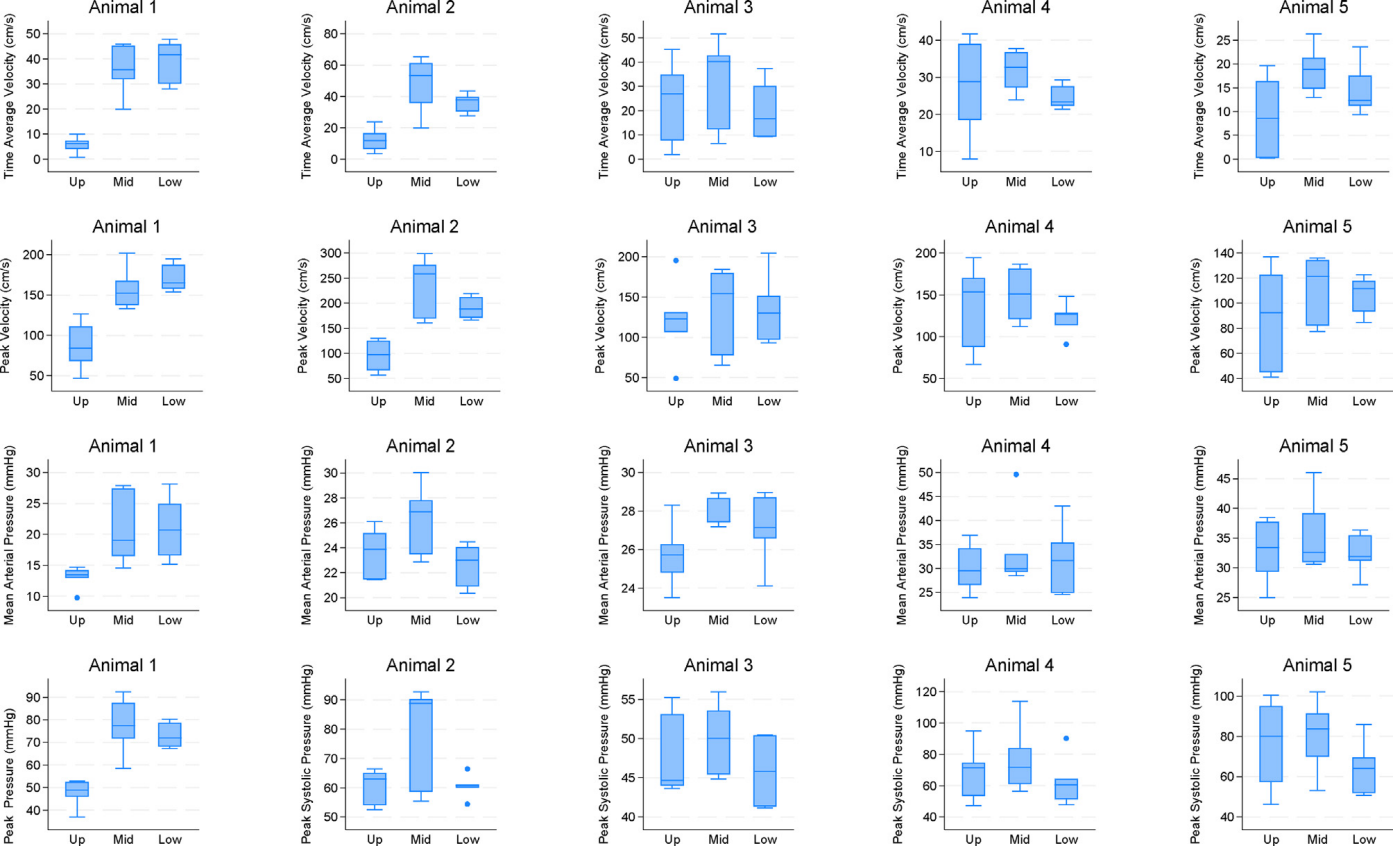
Boxplots of four outcome variables (Time Average Velocity, Peak Velocity, Mean Arterial Pressure and Peak systolic pressure) in five animals, according to the compression positions Upper (Up), Middle (Mid), and Lower (Low). Boxes represent the interquartile range (IQR) with the median value as the central mark in each box. Whiskers extend to the minimum and maximum data values, with any outliers beyond the whiskers indicated as dots.

Fig. 2 presents boxplots of TAV, peak velocity, MAP, and peak systolic pressure in all 5 animals according to chest compression position. The position with the highest TAV corresponded to the position with the highest MAP in three out of five animals. Furthermore, the position with the highest TAV corresponded with the position having the highest peak velocity in four out of five animals. Additionally, the position with the highest TAV matched the position with the highest peak pressure in four out of five animals. The findings highlight associations between TAV and MAP, TAV and peak velocity, as well as TAV and peak pressure in the studied animals.

A plot of the observed values along the sequential time is shown in Supplementary Fig. 2. Preliminary analysis of TAV revealed no systematic effect of either position order or sequence. The linear mixed model identified the middle position to have the highest expected TAV, seen in 4 out of 5 animals (Table 2). However, as may be observed in Fig. 2. and Table 2, we noted significant variability in TAV due to animal identity (SD = 9.7, 95% CI: 3.7 to 25.5), the slope of TAV along sequences within each animal (SD = 2.8, 95% CI: 1.3 to 6.1), and compression position within animal and sequence (SD = 8.4, 9 5% CI: 4.7 to 15.3). The latter estimate is of the same order as animal identity and residual (SD = 9.3, 95% CI: 7.8 to 11.0), emphasizing that one could not claim any best compression position overall.

By cross-tabulating the rankings of each single position (best, intermediate and worst) and the rankings of the overall positions within sequences, we found that the singly best positions matched the overall best position in 16 out of 29 sequences (55%). Similarly, the singly worst position matched the overall worst position in 20 out of 29 sequences (69%). Total mismatch (singly best = overall worst, and singly worst = overall best) was observed in 3 out of 29 (10%) and 1 out of 29 (3%) sequences, respectively (Supplementary Table 2). The number of sequences that were required to identify the optimal compression position differed among the animals (Supplementary Fig. 2).

## Discussion

In this animal study of cardiac arrest, we found that RescueDoppler yielded hands-free, real-time, hemodynamic CPR feedback to identify optimal as well as suboptimal chest compression positions. Chest compressions generated forward carotid blood flow with variations in TAV among different positions within each animal, across all animals.

Our results demonstrate that the guideline-recommended chest compression position yields the highest average TAV. However, even if a one-size-fits-all approach appeared effective for the majority of animals,^31^ the considerable variability observed suggests that it may not be uniformly effective across all individuals.

Previous human and animal research has shown the influence of anatomical variations on the hemodynamic effectiveness of chest compressions,^35^, ^15^, ^17^ and has shown that personalized hand placement guided by hemodynamic feedback can enhance outcomes in cardiac arrest.^3^, ^36^ Further, research has demonstrated that through measurements of carotid and femoral Doppler flow, the efficiency of chest compressions can be enhanced.^37^, ^38^, ^39^ Therefore, individualization of CPR techniques should be aligned with the anatomical differences of each patient.

By consistently and thoroughly monitoring chest compression quality through corrective feedback and real-time hemodynamic feedback, it might be feasible in the future to pinpoint optimal positions for compressions that yield the highest efficiency or avoid the least optimal position.

Identifying the least optimal position may be as important because it may prevent rescuers from administering compressions according to guidelines recommendations but compressing the least optimal position for the individual due to anatomical differences.^1^, ^4^, ^6^, ^17^, ^24^, ^25^ In this study, compressions were administered by a single experienced rescuer, ensuring a uniform technique throughout the experiment. Despite issues with accelerometer measurements, compression depth data from different positions exhibited minimal variation within each animal. Variability in the results was thus primarily caused by the compression position rather than the specific sequence within each animal and indicated that the rescuer consistently applied the same force or depth throughout the experiment.

Our study suggests that the use of RescueDoppler may be of help in identifying variations in TAV from altering chest compression positions. Further validation in clinical studies is necessary to prove the effectiveness of RescueDoppler as a hemodynamic feedback device to identify both optimal and suboptimal compression positions.

## Limitations

We acknowledge that the current study has several limitations. First, the study includes a small number size of animals. This may possibly reduce the generalizability of our findings, and the variance component estimates should be interpreted with some caution. Quantile-quantile plots of residuals and compression position as random effects did not suggest serious deviations from the assumptions of normality, however. Domestic pigs are proven models for cardiac arrest and CPR studies.^33^ Although pig-human differences don’t affect the RescueDoppler's function, human-specific calibration is necessary. The pig heart appears trapezoidal from the front.^40^ The heart shares apex and base regions with the human heart but with different upper and lower borders.^40^ The apex of the heart is formed entirely by the left ventricle.^40^ Its ventricular mass has a basic conical shape, with the anterior side against the sternum and the posterior side near the diaphragm.^40^ We chose three positions on the sternum for compressions, but other positions more lateral of the sternum could have been more efficient. The duration of chest compression at each position was limited to 10 seconds. However, this short time frame at each position might be less susceptible to interference from other factors during CPR. Furthermore, extending the duration of chest compressions at each position beyond 10 seconds could potentially yield more accurate estimations. Support for velocity changes due to position alterations is suggested by the homogeneity in animal characteristics, consistent application of compressions by an experienced rescuer, and the minimal impact of compression sequence on TAV variation, considering incomplete compression depth data for all animals. Although compression depth is a variable that could potentially affect the outcomes, compression depth data and our analysis did not suggest so.

The tissue movements resulting from the compressions introduced noise into the Doppler spectrogram. This noise poses a limitation for precise velocity calculations since the added wall and clutter filter filtered out low velocities.

## Conclusions

The use of RescueDoppler for hemodynamically guided CPR consistently detects alterations in blood flow velocity when changing the chest compression position in a porcine model. RescueDoppler has the potential to effectively pinpoint the chest compression positions associated with the highest and lowest carotid blood flow velocities.

## Funding

This project has received funding from Norwegian Research Council grant ID 324319, Norwegian University of Science and Technology grant ID 70443945, Nord University grant ID 03210275.

## CRediT authorship contribution statement

**Bjørn Ove Faldaas:** Writing – review & editing, Writing – original draft, Visualization, Methodology, Formal analysis, Conceptualization. **Erik Waage Nielsen:** Writing – review & editing, Visualization, Validation, Methodology, Formal analysis, Conceptualization. **Benjamin Stage Storm:** Writing – review & editing, Methodology, Investigation. **Knut Tore Lappegård:** Writing – review & editing, Resources, Methodology. **Bent Aksel Nilsen:** Writing – review & editing, Investigation. **Gabriel Kiss:** Writing – review & editing, Software. **Eirik Skogvoll:** Writing – review & editing, Visualization, Validation, Methodology, Formal analysis, Conceptualization. **Hans Torp:** Writing – review & editing, Validation, Software, Methodology, Investigation, Formal analysis, Data curation. **Charlotte Björk Ingul:** Writing – review & editing, Validation, Supervision, Resources, Project administration, Methodology, Investigation, Funding acquisition, Conceptualization.

## Declaration of competing interest

The authors declare the following financial interests/personal relationships, which may be considered as potential competing interests: Hans Torp and Charlotte Björk Ingul are employed by Cimon Medical which owns the technology associated with RescueDoppler.
