# Supplementary material for: Real-time feedback on chest compression efficacy by hands-free carotid Doppler in a porcine model
Source: Resusc Plus. 2024 Feb 20;18:100583. doi: 10.1016/j.resplu.2024.100583 (PMC10885784; doi:10.1016/j.resplu.2024.100583)
Supplement: Supplementary data 1 [file mmc1.pdf]

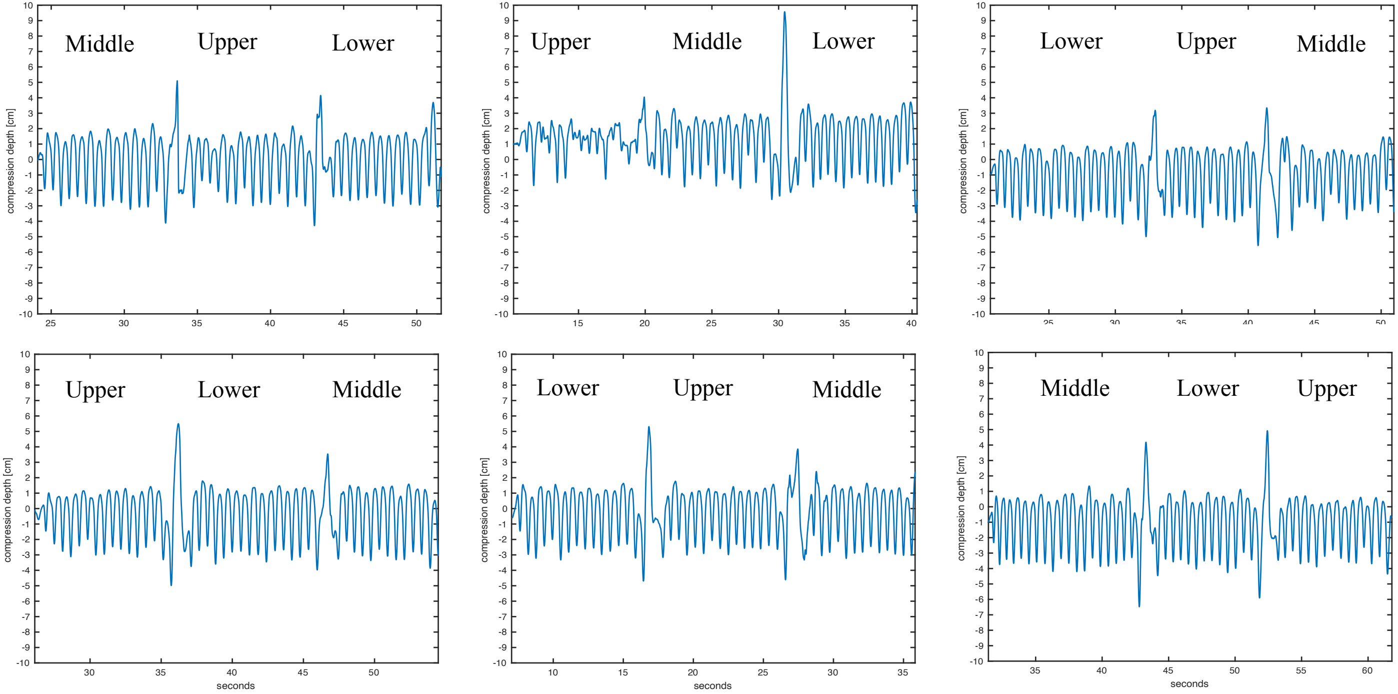

Supplement Fig. 1.

Compression depth data from a single animal illustrating six sequences. The accelerometer curve illustrates relative changes and compression depth is determined by measuring the distance between maxima and minima in the curve. Significant fluctuations in the curve occur when the accelerometer shifts between compression positions.
