# Supplementary material for: Real-time feedback on chest compression efficacy by hands-free carotid Doppler in a porcine model
Source: Resusc Plus. 2024 Feb 20;18:100583. doi: 10.1016/j.resplu.2024.100583 (PMC10885784; doi:10.1016/j.resplu.2024.100583)
Supplement: Supplementary data 2 [file mmc2.pdf]

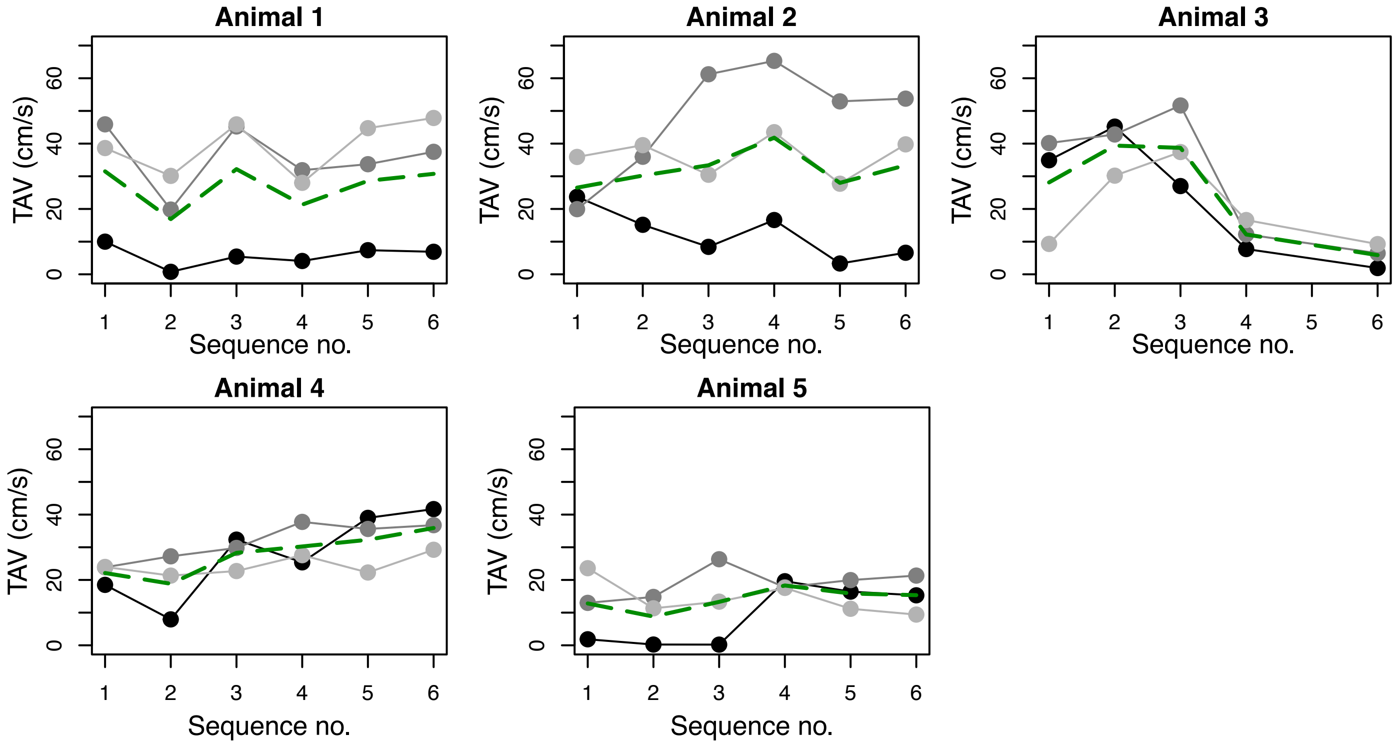

Supplement Fig. 3. Time Average Velocity (TAV) plot for sequence within animals.
TAV at each chest compression position is tracked over sequential time. X-axis= sequence number, Y-axis= TAV. Black dots: upper position, dark grey dots: Middle position, light grey: Lower position. The green dashed line represents the running mean value of TAV across all chest compression positions.
