# Supplementary material for: Real-time feedback on chest compression efficacy by hands-free carotid Doppler in a porcine model
Source: Resusc Plus. 2024 Feb 20;18:100583. doi: 10.1016/j.resplu.2024.100583 (PMC10885784; doi:10.1016/j.resplu.2024.100583)
Supplement: Supplementary data 3 [file mmc3.docx]

| **Supplement Table 1. Average compression depth (cm)** | | | | | | | | | | | |
| --- | --- | --- | --- | --- | --- | --- | --- | --- | --- | --- | --- |
|  | Animal 1 | | | | | | Animal 2 | | | Animal 3 | |
| **Sequence** | 1 | 2 | 3 | 4 | 5 | 6 | 1 | 2 | 3 | 3 | 4 |
| **Upper** | 4.0 | (!) | 3.8 | 4.1 | 4.2 | 4.1 | 3.8 | 3.7 | 3.5 | 3.0 | 3.3 |
| **Middle** | 4.7 | 4.1 | 4.3 | 4.2 | 3.8 | 4.3 | 3.0 | 4.0 | 3.8 | 3.0 | 3.6 |
| **Lower** | 4.0 | 4.1 | 4.0 | 4.1 | 3.8 | 4.2 | 4.6 | 4.2 | (!) | 3.7 | 4.0 |
| Average compression depth (cm) per animal and sequence from three of the five animals. Missing data are excluded or indicated by (!). | | | | | | | | | | | |
