# Supplementary material for: Real-time feedback on chest compression efficacy by hands-free carotid Doppler in a porcine model
Source: Resusc Plus. 2024 Feb 20;18:100583. doi: 10.1016/j.resplu.2024.100583 (PMC10885784; doi:10.1016/j.resplu.2024.100583)
Supplement: Supplementary data 4 [file mmc4.docx]

| **Supplement Table 2.** Cross-tabulation of chest compression positions by efficacy. | | | | |
| --- | --- | --- | --- | --- |
|  | **Overall Rank** | | | |
|  | B | I | W | Total |
| **Sequence Rank** |  |  |  |  |
| B | 16 | 10 | 3 | 29 |
| I | 12 | 11 | 6 | 29 |
| W | 1 | 8 | 20 | 29 |
| Total | 29 | 29 | 29 | 87 |
| Overall Rank; mean value of TAV over all chest compression and all sequences in each animal. Chest compression position ranked as best (B), intermediate (I) or worst (W) across all sequences (*Overall Rank, columns*) and within each sequence (*Sequence Rank, rows*). Example, first horizontal row: The sequence rank matched the overall rank on 16 occasions (B-B), differed by one on ten occasions (I-B), and a complete mismatch occurred on three occasions (B-W). | | | | |
